# Supplementary material for: Dynamic lattice distortions driven by surface trapping in semiconductor nanocrystals
Source: Nat Commun. 2021 Mar 25;12:1860. doi: 10.1038/s41467-021-22116-0 (PMC7994579; doi:10.1038/s41467-021-22116-0)
Supplement: Supplementary file 1 — Supplementary Information [file 41467_2021_22116_MOESM1_ESM.pdf]

Supplementary Information

Dynamic lattice distortions driven by surface trapping in semiconductor nanocrystals

Burak Guzelturk[1,2,#,*,†], Benjamin L. Cotts[1,†], Dipti Jasrasaria[3,†], John P. Philbin[3,†], David A. Hanifi[1,3], Brent A. Koscher[3,4], Arunima D. Balan[3,4], Ethan Curling[3], Marc Zajac[1], Suji Park[2], Nuri Yazdani[2,5], Clara Nyby[6], Vladislav Kamysbayev[7], Stefan Fischer[1], Zach Nett[3], Xiaozhe Shen[8], Michael E. Kozina[8], Ming-Fu Lin[8], Alexander H. Reid[8], Stephen P. Weathersby[8], Richard D. Schaller[9,10], Vanessa Wood[5], Xijie Wang[8], Jennifer A. Dionne[1], Dmitri V. Talapin[7,9], A. Paul Alivisatos[3,4,11,12], Alberto Salleo[1], Eran Rabani[3,4,13], Aaron M. Lindenberg[1,2,6,14,*]

Contents

Supplementary Figures S1-18
Supplementary Table 1
Supplementary Methods
Supplementary References

Section A: Synthesis and sample prep:

High quality samples of CdSe and CdSe/CdS were used from the same batches detailed in Ref[1]. CdSe cores were prepared via a hot injection synthesis. The average diameter of the core is 3.5nm from optical sizing. CdSe/CdS core/shell particles were prepared with a further overgrowth of 8 monolayers of CdS onto cores via a continuous slow injection shell growth. Full synthetic and characterization details for the quantum dot stock solutions can be found within Ref[1].

Aliquots of quantum dot solution stored in hexanes were washed via centrifugation with methyl acetate as the antisolvent to remove excess ligand and resuspended in toluene for casting onto TEM grids. Films of ~1-3 monolayers were screened in a FEI Tecnai Transmission Electron Microscope for clear electron diffraction before loading at SLAC MeV-UED. Films were also imaged following measurements at SLAC MeV-UED facility to ensure no sintering or other irreversible transformations took place. Representative images of the films are shown in Supplementary Figure 1. Absorbance of the samples are also shown in Supplementary Figure 2.

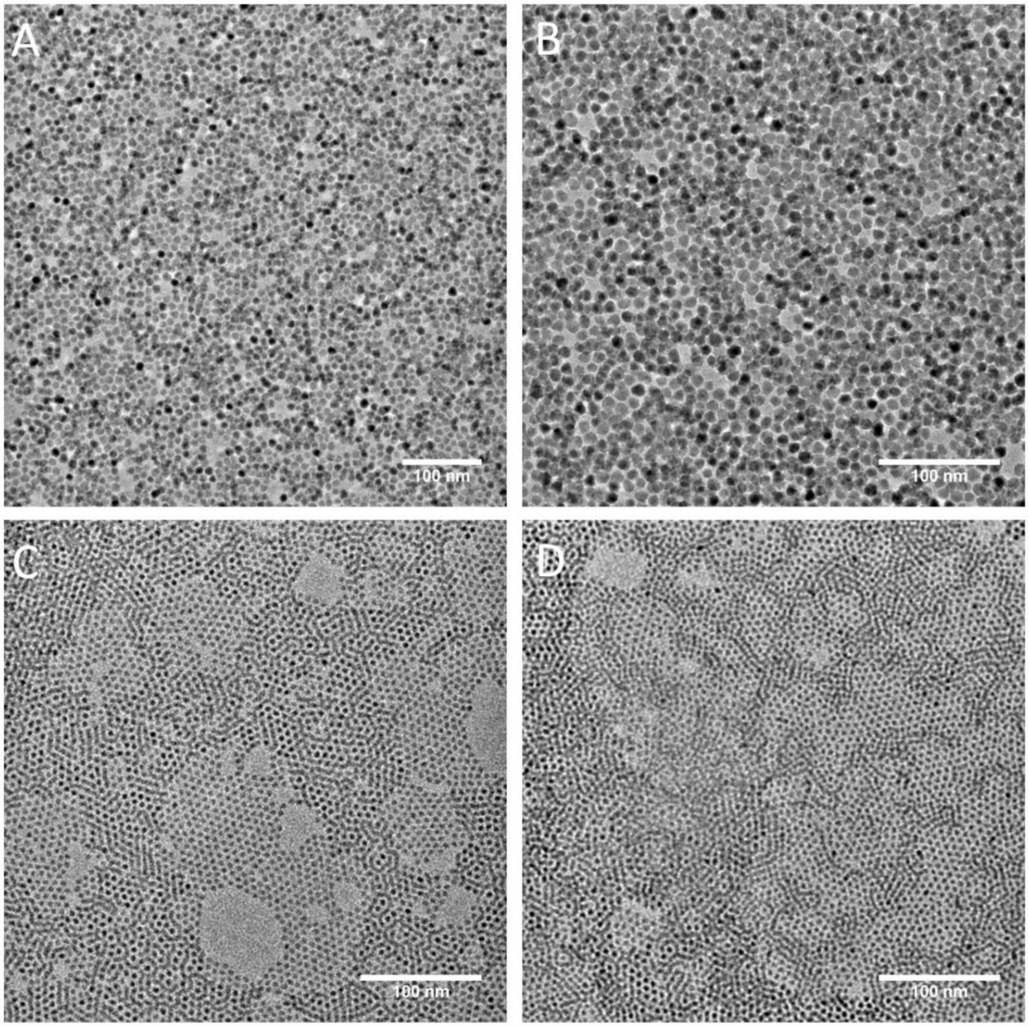

Supplementary Figure 1. Transmission electron microscopy images of the core/shell CdSe/CdS nanocrystal with 8 monolayer-thick shell (A,B), CdSe core-only nanocrystal (C, D).

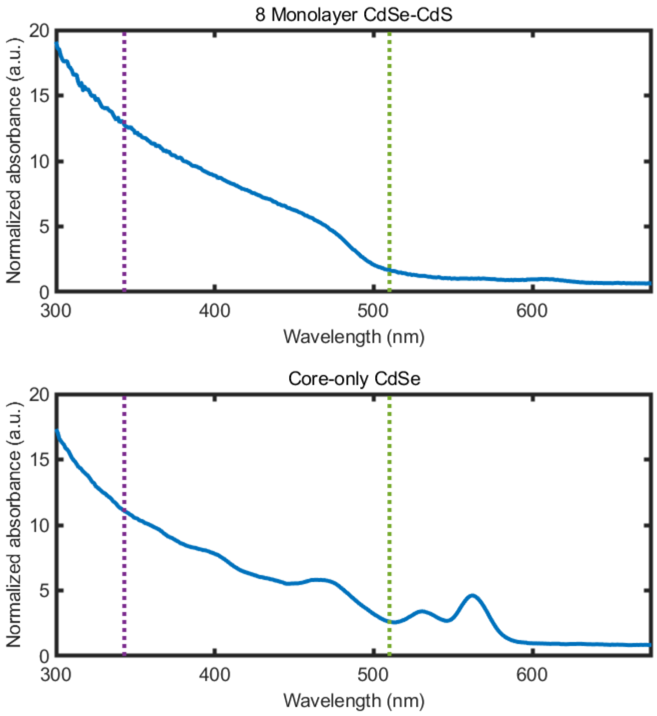

Supplementary Figure 2. Absorbance of the core/shell and core-only nanocrystals. Dashed lines indicated pump laser wavelengths at 343 nm (purple) and 510 nm (green). Note that the bulk CdS bandgap onset is at ~512 nm, and as such, the 510 nm excitation predominantly excites the core of the nanocrystals.

Section B. UED data collection and analysis

UED data was collected at the SLAC MeV-UED instrument, part of the LCLS User Facility. Details of the system have been reported before[2]. A multipass Ti:sapphire laser (800nm, 60 fs, 360 Hz) is split into beam paths serving as both the optical excitation source and to generate pulsed electron bunches from the photocathode. For generating the electron probe, a portion of the laser output is frequency tripled. The resultant electron beam pulses are accelerated to 3.7 MeV (~200 fs FWHM) for 50 fC per pulse. The other portion of the laser output is fed to an optical parametric amplifier to generate the two varied pump energies (340 or 510 nm). The size of the electron probe was ~100 µm, and the pump beam was ~ 500 µm to ensure good spatial overlap. The diffracted electrons were detected via a red P43 phosphor screen and captured with an Andor iXon Ultra 888 EMCCD detector. Time zero was calibrated for pump-probe translational delay stage position using the sharp response from either thin single-crystal silicon or bismuth. Electron diffraction images were integrated for 10 seconds at each time delay position. Delay positions were collected in randomized order to minimize systematic effects, with the order re-randomized between each scan.

Diffraction images were analyzed similar to that in our previous work[3]. Images were background subtracted for CCD dark counts and hot pixels were removed, following beam centering the images were radially integrated to calculate $I(Q)$ for each image. Images were sorted by pump-probe delay labels and scans were averaged together to get $I(Q,t)$. Predefined peak areas were fit with a local linear background

and peak intensity over time was analyzed. In Figure 2A, we plot $\frac{I(Q,t)}{I_0}$, where I_0 is the intensity of the before time zero, in the absence of the pump beam.

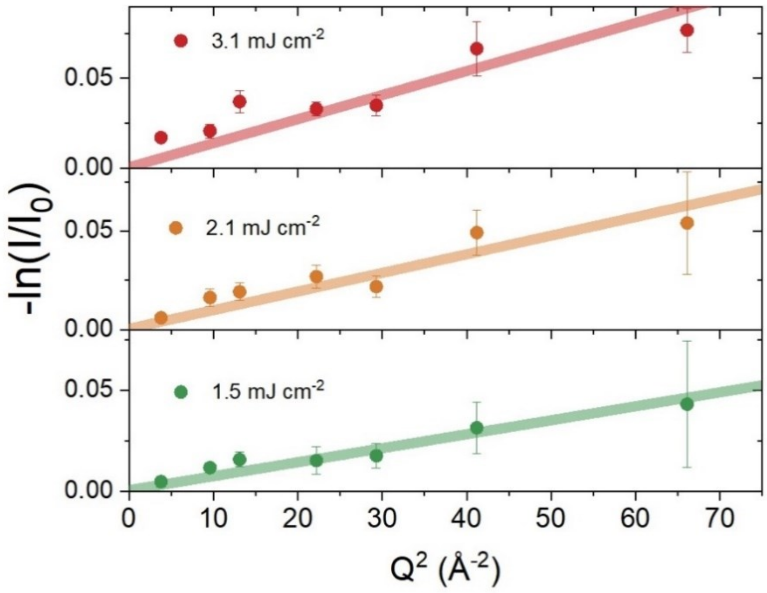

Supplementary Figure 3. $-\ln(I(t)/I_0)$ plotted as a function of Q^2 for different excitation fluences under 510 nm excitation. All fluence cases show a linear response confirming that the transient effect arises from a Debye-Waller (DW) effect. The error bars show standard error.

Supplementary Table 1: Major contributing reciprocal planes for each Q peak cited in the main text (Fig. 1b). Due to the sample-detector distance chosen to maximize q range and the overlapping contributions from CdS and CdSe, the peaks are not separately distinguished. Some minor peaks are omitted.

	Q1	Q2	Q3	Q4	Q5	Q6	Q7
Major contributing reciprocal planes	(100)<br>(002)<br>(101)	(110)	(112)	(203)<br>(210)<br>(211)	(213)<br>(302)	(215)<br>(116)<br>(222)	Unassigned*

*beyond reported range in ICDD tables [PDFs 000-041-1049 and 00-008-0459][4]

<u>Section C. Transient atomic pair distribution function analysis</u>

As described in Ref[3,5,6], we calculate the transient differential atomic pair distribution function, $\Delta G(r,t)$ to examine the transient evolution of the atomic pair correlations as a function of pump-probe delay. First the differential diffraction intensity $\Delta I(Q,t) = I(Q,t) - I_0(Q)$ is calculated for delay times after time zero. $I_0(Q)$ is the diffraction intensity before time zero. We then calculated the differential total structure function, $\Delta S(Q,t) = \frac{\Delta I(Q,t)}{|f|^2}$. Here, f is the atomic form factor:

$$f = \sum_j a_j e^{(-b_j s^2)} + \frac{m_0 e^2}{8\pi^2 \hbar^2}\left(\frac{\Delta Z}{s^2}\right)$$

and we used the relation between Q and s, $s = \frac{\sin(\theta)}{\lambda} = \frac{Q}{4\pi}$. We obtained a and b coefficients for the parametrized atomic form factor from Ref[7]. Finally, we perform a Sine Fourier transform to convert to the transient differential pair distribution function, $\Delta G(r,t) = \frac{2}{\pi}\int_{Q_{min}}^{Q_{max}} Q * \Delta S(Q,t) * \sin(Qr)\, dQ$.

Section D. Molecular dynamics simulations of CdSe/CdS nanocrystals

Nanostructure configurations for CdSe cores were obtained by cleaving a sufficiently large wurtzite crystal with a lattice constant of bulk wurtzite CdSe ($a = 4.30$Å$, c = a\sqrt{8/3}$) such that all Cd and Se atoms are bonded to at least two other atoms. To obtain core-shell structures, monolayers of CdS were added to the CdSe core structure. The CdSe core has configuration $Cd_{462}Se_{462}$ and a diameter of 3.9nm. The core-shell with four monolayers (ML) of CdS shell has configuration $Cd_{2637}Se_{483}S_{2154}$ and a total diameter of 7.3nm. The core-shell with 8 ML of CdS shell has configuration $Cd_{7872}Se_{483}S_{7389}$ and a diameter of 10.3nm.

All molecular dynamics (MD) simulations were performed using the LAMMPS code[8] and a previously implemented potential with Lennard Jones and Coulomb terms that is parameterized for CdSe and CdS[9].

Computing pair distribution functions from MD

To compute $\Delta G(r, \Delta T = 14\text{ K})$, which is referenced in the main text and illustrated in Fig. 2C of the main text, the generated core/shell nanocrystals (with 8 ML shell) are first equilibrated at 300 K and 314 K using the Langevin thermostat implemented in LAMMPS. The radial distribution function ($g(r)$) is computed directly using LAMMPs from the equilibrium trajectory at each temperature and then transformed to the atomic pair distribution function (PDF):

$$G(r) = 4\pi\rho_0 r[g(r) - 1],$$

where ρ_0 is the average number density. The $G(r)$ at each temperature is then smoothed using a moving average technique with a smoothing window of 0.14 Å. Finally, the differential atomic PDF is computed by subtracting the atomic PDF at each temperature:

$$\Delta G(r, \Delta T = 14\text{ K}) = G(r, T = 314\text{ K}) - G(r, T = 300\text{ K}).$$

Computing relationship between Debye Waller factor and mean square atomic displacement

For a given temperature, the Debye Waller factor (DWF) can be computed at different Q values using an equilibrium MD trajectory. It can be computed according to different assumptions. Under the assumption of harmonic and isotropic atomic motion, the DWF is given by

$$\text{DWF}_{\text{h,i}} = \exp\left(-\frac{Q^2\langle u^2\rangle}{3}\right),$$

where $\langle u^2\rangle$ is the mean square displacement (MSD). When the atomic motion is assumed to be harmonic but not isotropic, the expression for the DWF is given by

$$\text{DWF}_{\text{h}} = \exp(-\langle \boldsymbol{Q}\cdot\boldsymbol{r}\rangle^2).$$

Finally, when there are no assumptions regarding the atomic motion, the DWF is calculated as

$$\mathrm{DWF} = \exp(\langle i\boldsymbol{Q} \cdot \boldsymbol{r} \rangle)^2.$$

For the core/shell structures (with 8 MLs of shell), the DWF was computed according to these three different expressions using the atomic positions from an equilibrium MD trajectory at 300 K. For the examined range of Q values (Supplementary Figure 4), the computed DWF are consistent with one another, even when the assumptions of harmonic and isotropic atomic motion are relaxed.

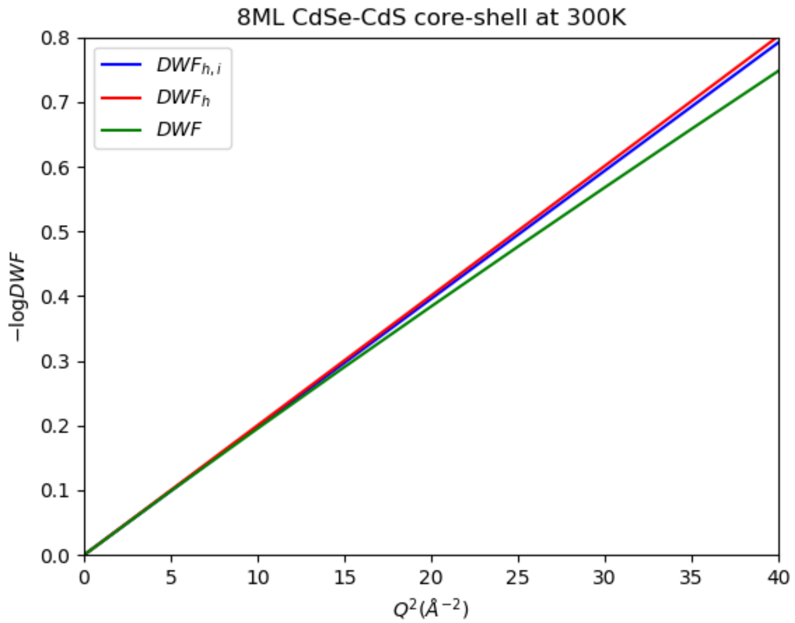

Supplementary Figure 4. The $-\log DWF$ (DWF = Debye Waller Factor) computed for a core/shell nanocrystal from an molecular dynamics (MD) simulation at 300 K under the three different levels of assumption described above. We see that the deviation between the DWFs calculated at different levels of approximation is minimal over this range of Q values, illustrating that the use of the experimental DWF to compute mean square atomic displacements ($\langle u^2 \rangle$) is justified for these systems, even though the atomic motion is not necessarily harmonic and/or isotropic.

The MSD was computed from equilibrium MD trajectories at different temperatures (150K, 300K, 310K, 320K, 400K, and 500K) for the core-shell structure (with 8MLs of shell). The relationship was found to be linear, as illustrated in Supplementary Figure 5, with a slope of 0.000223 Å/K. This slope matches literature values for similar systems[10] and was used to compute the temperature rise of the nanocrystal system after optical excitation, as indicated in the main text.

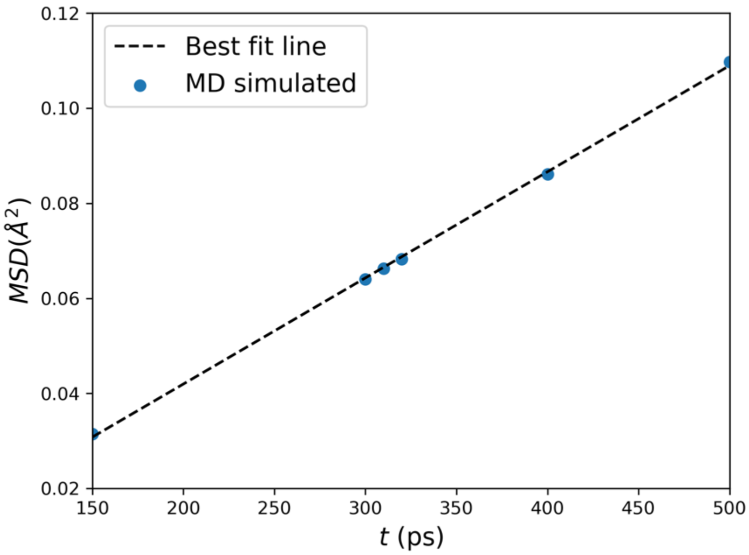

Supplementary Figure 5. Mean squared atomic displacements (MSD) calculated by MD simulations for the core/shell structure at temperatures ranging from 150 K to 500 K. The relationship between the temperature and mean square displacement is linear, and the slope of the data gives the Debye-Waller factor used in estimating the transient lattice temperature of the samples in the main text.

Computing the phonon density of states and contribution of phonon modes to Debye Waller factor

MD simulations were used to compute the phonon density of states and phonon lifetimes for a core/shell structure (with 4 MLs of shell). First, the core/shell structure was minimized using the conjugate gradient descent algorithm implemented in LAMMPS to obtain the zero-temperature structure. Using this configuration, the mass-weighted Hessian (i.e. the second derivative of the potential with respect to atomic coordinates) was computed and diagonalized, yielding the phonon frequencies and modes as the eigenvalues and eigenvectors, respectively. These phonon frequencies are illustrated as the phonon density of states in Supplementary Fig. 6. Aside from the six zero-frequency modes, which correspond to the translational and rotational degrees of freedom of the nanocrystal, the lowest-frequency mode has a frequency of 0.17 THz, indicating that there is some confinement of the bulk phonon modes, but it is not significant.

To estimate the contribution of different phonon modes to the Debye Waller factor. We consider the following[11]:

$$DWF \propto \sum_{kj} \frac{1}{\omega_p(kj)} \coth\left(\hbar\omega_p(kj)\right)$$

ω_p is the phonon frequency. At low frequencies (near the zone center where k ≈ 0), the hyperbolic cotangent can be approximated as $\frac{1}{\omega_p}$. Thus, we can approximate the contribution of different frequency mode phonons to DWF as:

$$DWF \propto \sum_{kj} \frac{1}{{\omega_p}^2(kj)}$$

To find the contribution term, we multiply the calculated phonon density of states with the $\frac{1}{{\omega_p}^2}$ as shown in Supplementary Figure 6.

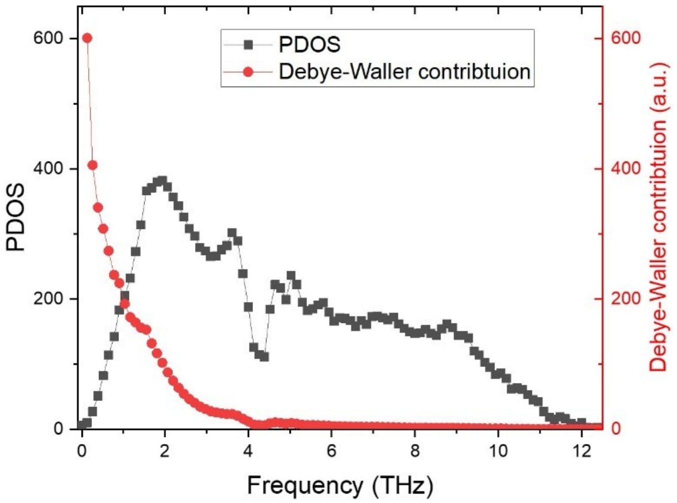

Supplementary Figure 6. Computed phonon density of states (black) and the contribution of phonon modes to the Debye-Waller factor (red) as a function of phonon frequency. Smaller frequency acoustic phonons have a larger contribution to the Debye-Waller factor due to their larger contribution to mean squared atomic displacements.

Computing phonon lifetimes

The phonon lifetimes, *i.e.* the T_1 timescale of relaxation of a populated phonon mode, which are referenced in Figure 2E of the main text, were computed within linear response for each phonon mode[12–14]. To obtain the relaxation lifetime of each phonon mode, the core/shell structure (with 4 MLs of shell) was equilibrated at 300 K using a Langevin thermostat and then a trajectory in the microcanonical ensemble was simulated. The calculated phonon modes were then used to transform the positions and velocities of the trajectory from atomic coordinates ($x(t)$ and $v(t)$) to phonon mode coordinates ($q(t)$ and $\dot{q}(t)$).

The motion of a tagged phonon mode can be described by a generalized Langevin equation:

$$\ddot{q}(t) = -\omega^2 q(t) + R(t) - \int_0^t dt' \xi(t-t')\dot{q}(t'),$$

where ω is the frequency of the mode, $R(t)$ is the Langevin force, and $\xi(t)$ is the friction kernel. As derived in the references cited above, the relaxation timescale of the mode can be calculated as

$$T_1^{-1} = \tilde{\xi}'(\omega),$$

where $\tilde{\xi}'(\omega) = \Re\left[\int_0^\infty \xi(t)e^{-i\omega t}dt\right]$ is the real part of the Laplace transform of the friction kernel at the frequency of the mode.

Since there are no assumptions being made about the nature of the coupling between modes, the friction kernel for each mode can be obtained using the velocity autocorrelation function of the mode,

$$C(t) \equiv \langle \dot{q}(t)\dot{q}(0) \rangle.$$

The Laplace transform of the friction kernel at the frequency of the mode can then be calculated directly from the Laplace transform of the velocity autocorrelation function:

$$\tilde{\xi}'(\omega) = \Re\left[\frac{C(0) - i\omega\tilde{C}(\omega)}{\tilde{C}(\omega)} + i\omega\right].$$

This relation can be derived by writing the generalized Langevin equation above in terms of the velocity of the mode $\dot{q}(t)$, multiplying the equation by the initial velocity $\dot{q}(0)$, taking the average of both sides with respect to initial conditions, and performing a Laplace transform of both sides.

The velocity autocorrelation function for each mode was computed by averaging trajectories over different initial conditions. As illustrated in Supplementary Figure 7, the velocity autocorrelation function features an initial decay followed by long-lived beatings that arise from the anharmonic coupling between modes. Note that the velocity autocorrelation functions were also computed for the core/shell structure at 1 K. At this low temperature, the correlation functions oscillated between 1 and -1 and do not decay, suggesting pure harmonic atomic motion, which is expected at such low temperatures.

The numerical Laplace transform of each velocity autocorrelation function was obtained. To allow for convergence of these numerical Laplace transform, the velocity autocorrelation functions were multiplied by a Gaussian factor ($C(t) \approx C(t)\exp(-\eta t^2)$) with a dampening parameter η that allowed for the correlation function to reach a value of 10^{-16} by 200 ps. The calculated phonon lifetimes are converged with this choice of dampening parameter. Finally, the Laplace transform of the velocity autocorrelation function was used to calculate the Laplace transform of the friction kernel, according to the equation given above, and the real part of the Laplace transform of the friction kernel was related to the phonon lifetime.

This MD approach for computing phonon lifetimes was verified for the simple model of a tagged particle bilinearly coupled to a harmonic bath, which can be solved analytically. While the phonon lifetimes are computed within linear response, *i.e.* in the regime of a mode being slightly perturbed, we would expect the lifetime to be even shorter for response from a larger perturbation as well as for higher temperatures. Furthermore, while these results were computed for a core-shell structure with 4ML shell, we believe that the lifetimes would not change significantly for an 8ML shell structure, for which the computational cost of computing and diagonalizing the Hessian was prohibitive. Calculations of phonon lifetimes on just a core structure at 300 K were comparable to the lifetimes for the 4 ML core/shell structure, with the vast majority of modes having sub-picosecond lifetimes and the lowest-frequency modes having lifetimes around 7 ps. Thus, the phonon lifetimes for the core only nanocrystal are similar to those of the 4 ML core/shell nanocrystal and suggest that the phonon lifetimes the 8 ML core/shell should be very similar to those presented in main text Figure 2E.

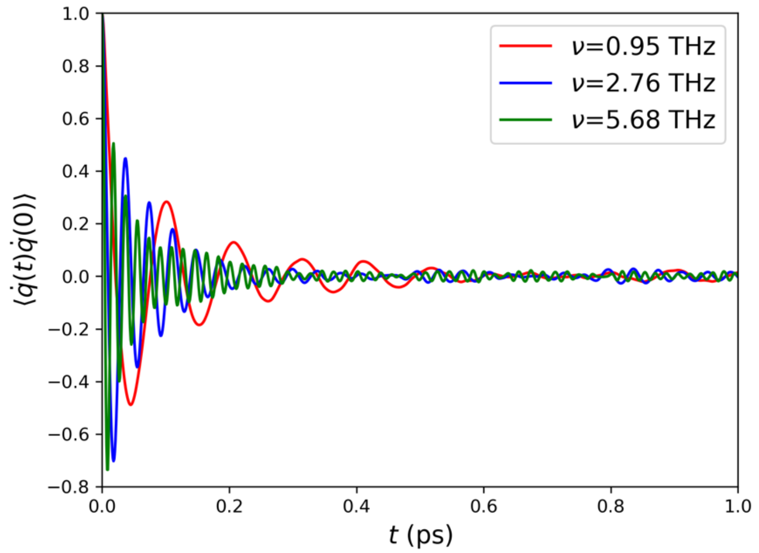

Supplementary Figure 7. Velocity autocorrelation functions for three modes computed for the 4ML core-shell structure using MD simulations. As expected, higher frequency modes decay more quickly. For all modes, the initial fast decay is followed by long-lived beatings due to anharmonicities in the system.

Section E. Energy coupled to lattice from photoexcitation to increase temperature by 1 K

In main text Figures 2D and 3D, the left y-axis is transduced from the relative decrease in the diffraction peak intensity via the Debye-Waller factor (DWF) as detailed above. The righthand y-axis is expressed in units of energy gained (ΔE) by the lattice in units of eV. Here we detail this mapping via per particle specific heat for a temperature rise of 1 K. The volume of the particle is $V_{particle} = \frac{4}{3}\pi r^3$, where *r* is the radius of the nanocrystal. We consider the specific heat of the CdSe/CdS nanocrystals by considering the volume ratio of the core and the shell: $V_{shell} = V_{particle} - V_{core}$. Specific heat of the core/shell (8 ML CdS) sample is $C_{8ML} = ((\rho_{CdSe} * V_{core}) * C_p^{CdSe,g}) + ((\rho_{CdS} * V_{shell}) * C_p^{CdS,g})$. The size of the core is 3.5 nm and the width of each shell layer is expected to be ~.3 nm. These volumes are combined with the CdSe and CdS densities and gravimetric specific heat to calculate the per particle specific heat for the 8ML core-shell particle. The energy needed to increase the temperature of the nanocrystal by 1 K ($= \Delta T$) is equal to $\Delta E = C_{8ML} \times \Delta T$. We estimate ΔE to be ~4 eV.

Section F. Kinetic models (Auger heating model and hot hole surface trapping) and time-resolved photoluminescence measurements

In order to decipher the time dependence of nanocrystal heating (main text Figs. 2D and 3D), we developed a kinetic model that accounts for the timescales of the different decay pathways of electron-hole pairs in CdSe/CdS core/shell nanocrystals (NCs). The important energy scales for these NCs are displayed in Supplementary Figure 8. The specific decay pathways of the photogenerated electron-hole pairs are hot (i.e. high energy) carrier cooling to the band-edge, Auger recombination (similar to exciton-exciton annihilation), hole trapping, and radiative recombination. Because the radiative recombination lifetime of excitons is ~45 ns in these NCs[1], it has a negligible impact on the dynamics of the photogenerated electron-hole pairs on the sub-nanosecond timescale of these experiments. Hot carrier cooling, Auger recombination, and hole trapping are shown schematically in Supplementary Figure 9. Importantly, hot carrier cooling is

the only aforementioned event that results in heating of the lattice as phonons are emitted throughout the cooling process.

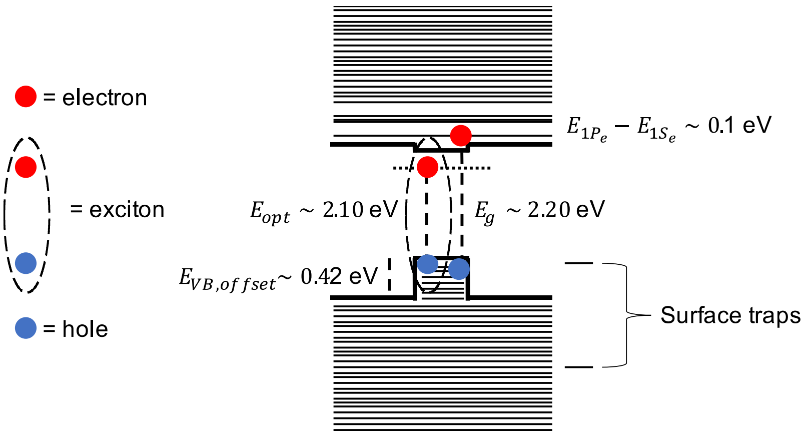

Supplementary Figure 8. An energy level diagram for the CdSe/CdS core/shell quantum dot studied in this work with important energy scales labelled.

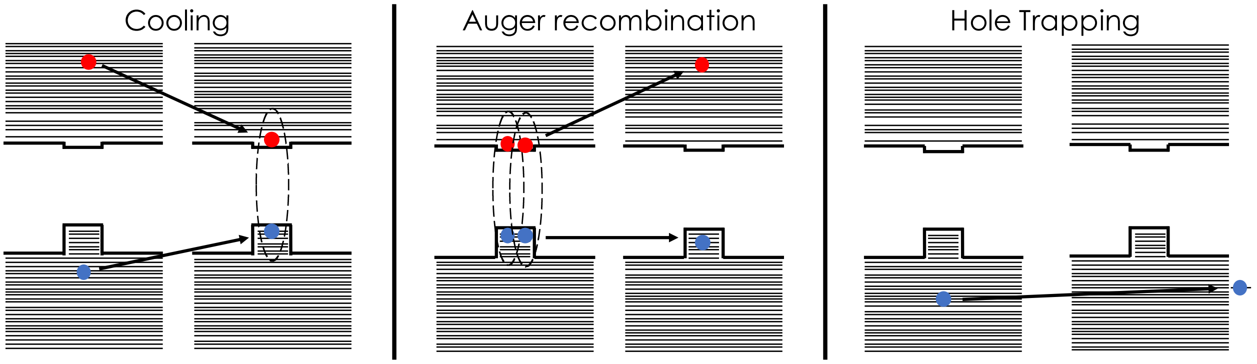

Supplementary Figure 9. Schematic showing the relevant electron-hole pair decay pathways in CdSe/CdS core/shell quantum dots. These events make up the events in the kinetic model. The left panel shows carrier cooling (which is accompanied by phonon emission). The middle panel shows an Auger recombination for which the electron receives a majority of the energy from the recombining electron-hole pair. The right panel shows a hole trapping event.

Kinetic model for Auger heating (Figure 2D in the main text)

We begin by discussing the kinetic model with respect to the low photon energy, 510 nm optical pump before describing how it must be modified to explain the high photon energy, 340 nm optical pump ultrafast electron diffraction data. The low energy optical pump generates electron-pairs that are near the band-edge of the CdSe/CdS core/shell NC. This is because there is a large (~420 meV) valence band offset between CdSe and CdS (Supplementary Figure 8). In other words, immediately following the optical pump, there are many electron-hole pairs (i.e. excitons) located on the CdSe core of the CdSe/CdS core/shell NC. The left panel of Supplementary Figure 10 shows this pictorially for an optical pump that generates four initial electron-hole pairs. In our kinetic model, the number of electrons (n_{e}), holes (n_{h}), and, thus, excitons (n_{exc}) generated by the optical pump is the initial condition and, as expected, increases linearly as the pump fluence increases. Because these electrons and holes are generated close to the band-edge, they do not

initially possess significant if any excess energy to release to the lattice via phonon emission. However, because there are multiple electron-hole pairs (i.e. excitonic states), Auger recombination can generate hot electrons (n_e^{Auger}) and holes (n_h^{Auger}) which can then cool back to the band-edge, thereby heating the lattice. This process is termed Auger heating and is shown schematically in Supplementary Figure 10. We hypothesized that the timescale of lattice heating for the low photon energy, 510 nm optical pump, which occurs on the order of a few hundreds of picoseconds is controlled by the timescale of Auger recombination in these CdSe/CdS core/shell NCs.

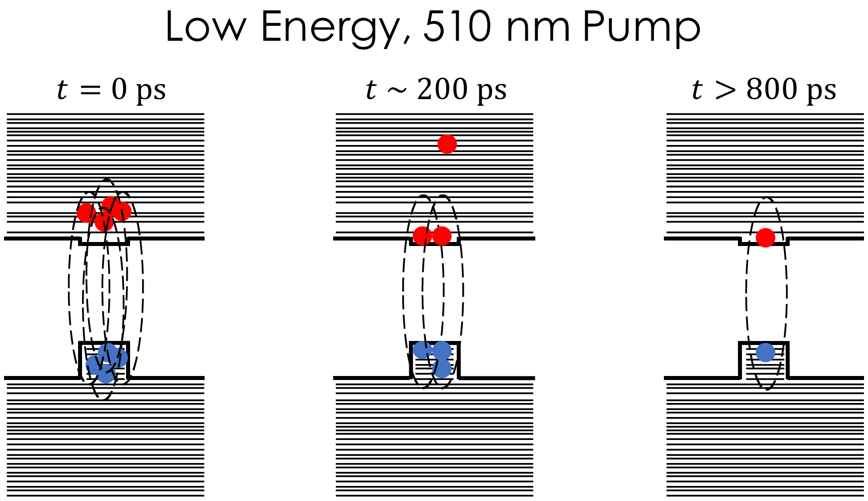

Supplementary Figure 10. Schematic representation of the initial state (left), an intermediate state (middle), and final state (right) of the kinetic model for the low energy, 510 nm pump. The intermediate state shows that a high energy electron has been generated by an Auger recombination event. It is the cooling of this high energy electron that results in lattice heating.

Before showing the results of our kinetic fits, we will outline the mathematics of the model and describe the fitting process. Mathematically, the model for the low energy, 510 nm pump consists of a set of coupled differential equations:

$$n_{exc} = \min(n_e, n_h) \quad (S1)$$

$$\frac{dn_e}{dt} = -\frac{n_{exc}(n_{exc}-1)}{2} k_{Auger} + n_e^{Auger} k_{cool} \quad (S2)$$

$$\frac{dn_h}{dt} = -\frac{n_{exc}(n_{exc}-1)}{2} k_{Auger} + n_h^{Auger} k_{cool} \quad (S3)$$

$$\frac{dn_e^{Auger}}{dt} = \frac{n_{exc}(n_{exc}-1)}{2} k_{Auger} - n_e^{Auger} k_{cool} \quad (S4)$$

$$\frac{dn_h^{Auger}}{dt} = \frac{n_{exc}(n_{exc}-1)}{2} k_{Auger} - n_h^{Auger} k_{cool} \quad (S5)$$

$$\frac{dT_{lattice}}{dt} \propto n_e^{Auger} k_{cool} + n_h^{Auger} k_{cool} \quad (S6)$$

where n_{exc} is the number of low energy excitons, $n_{\mathrm{e(h)}}$ is the number of electron (holes) near the band-edge, $n_{\mathrm{e(h)}}^{\mathrm{Auger}}$ is the number of hot (i.e. high energy) electrons (holes) that are produced from Auger recombination events as shown in Supplementary Figure 9. Eqs. S1-S6 were solved using the Gillespie algorithm[15], which is a trajectory-based algorithm. This Markov chain Monte Carlo algorithm begins with an initial distribution of electron-hole pairs in each NC that is given by a Poisson distribution with the average number of initial electron-hole pairs being determined by the pump fluence and the absorption cross section at the given pump energy. Specifically, our initial conditions are $\langle n_{\mathrm{exc}}(t=0)\rangle = \langle n_{\mathrm{e(h)}}(t=0)\rangle \propto \sigma J$ where σ is the absorption cross section at the given pump wavelength, J is the pump fluence, and $\langle\ \rangle$ denotes an average and $\langle n_{\mathrm{e(h)}}^{\mathrm{Auger}}\rangle = 0$. The prefactors ($n_{\mathrm{e(h)}}^{\mathrm{Auger}}$ and $\frac{n_{\mathrm{exc}}(n_{\mathrm{exc}}-1)}{2}$) in front of k_{cool} and k_{Auger} arrive from statistical scaling arguments[16]. In these systems, we know that electron-hole pairs form bound Wannier excitonic states with exciton binding energies on the order of 100s of meV. Thus, free-carrier approximations are not valid, and we treat excitons as the fundamental quasiparticle in our kinetic models. We assume that cooling is unimolecular process and that Auger recombination is a bimolecular process to obtain the aforementioned prefactors. Therefore, the only parameters that need to be determined in our Auger heating kinetic model are the single exciton cooling rate (k_{cool}) and the biexciton Auger recombination rate (k_{Auger}).

Cooling of a hot electron-hole pair to the band-edge is known to occur on the order of a picosecond timescale[17]. Thus, we set the single exciton cooling rate to be fast ($k_{\mathrm{cool}} = (2.0\ \mathrm{ps})^{-1}$), and we note that changing this fast timescale by a factor of two in either direction does not qualitatively change the results (and quantitative changes are limited to only a few percent). Auger recombination lifetimes ($\tau_{\mathrm{Auger}} = k_{\mathrm{Auger}}^{-1}$) of NCs are typically measured via either transient absorption or time-resolved photoluminescence experiments. To this end, we performed time-resolved photoluminescence experiments (Supplementary Figure 11) using a 510 nm pump to initially excite the NCs and fit the photon counts as a function of time using Eqs. S1-S6, under the assumption that the photon counts at a given time are proportional to the average number of excitons at the same time. The results of this fitting process gave us a biexciton Auger recombination lifetime of ~625 ps. The excellent agreement between our model and the experiments for all pump fluences are shown in the right panel of Supplementary Figure 11. Furthermore, this Auger recombination lifetime is very reasonable for these NCs and is in agreement with past experimental and theoretical studies[18,19].

To obtain main text Figure 2D, we simply applied Eqs. S3-S8 with the aforementioned cooling and Auger recombination rates and monitored the temperature change of the lattice as a function of time (Eq. S6). It is immediately evident from main text Figure 2D that the kinetic model describes the kinetics of lattice heating for the low energy, 510 nm pump very well. Importantly, the timescale of heating of the lattice for this pump fluence is determined by the timescale of biexciton Auger recombination along with the average number of initial excitons due to the approximately quadratic scaling of the total Auger recombination rate with the number of excitons on the NC. The time dynamics for the 510 nm pump are summarized pictorially in Supplementary Figure 10.

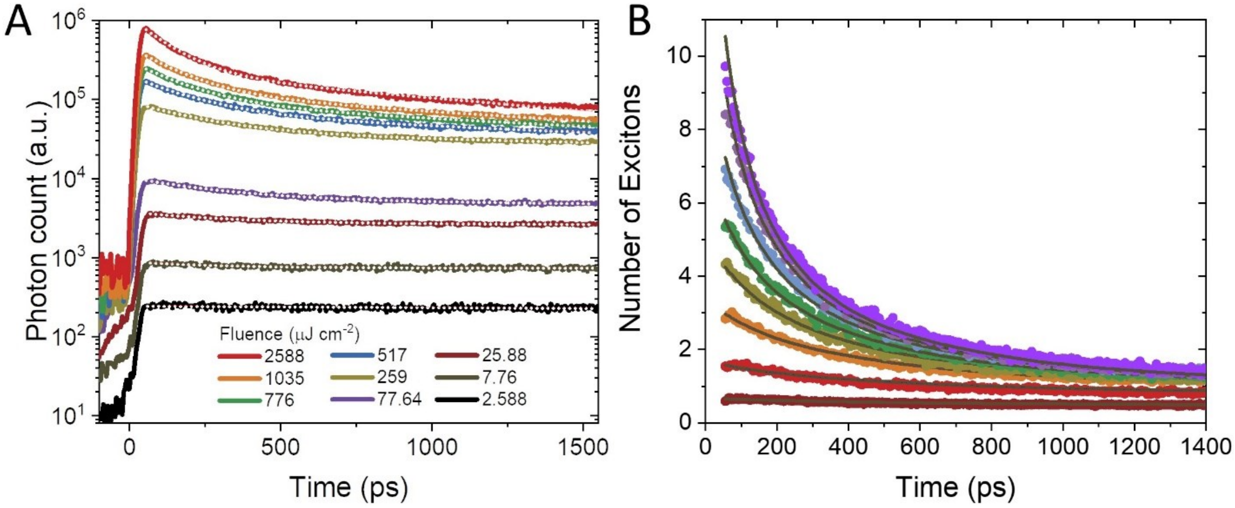

Supplementary Figure 11. (A) Transient photoluminescence decay dynamics for 8ML core-shell measured in hexanes solution and excited at 510 nm and detected using a streak camera. Biexponential fits are displayed in dashed lines, used to extract the k_{Auger} lifetime. (B) Intensities scaled to a number of excitons for each fluence along with kinetics fits using Eqs. S1-S6 (solid lines).

Kinetic model for hot hole surface trapping and Auger heating (Figure 3D in the main text)

Interestingly, the same kinetic equations (Eqs. S1-S6) fail to capture the dynamics of heating for the high energy, 340 nm pump (as shown in main text Figure 3D). Specifically, the overall heating is on a much faster timescale for the high energy pump (tens of picoseconds as opposed to hundreds picoseconds) and the magnitude of heating is much less than expected given that the absorption cross section is a factor of ~5 larger at 340 nm than at 510 nm for these NCs, which results in the average number of initial excitons being a factor of ~5 larger for the high energy pump. To remedy this failure along with accounting for the localized structural disorder that is present in the 340 nm pump ultrafast electron diffraction data, we added a hole trapping process (right most panel of Supplementary Figure 9) that occurs on a picosecond timescale and results in the trapping of ~80% of the initial holes. Additionally, we added a negative trion Auger recombination process to account for the fact that there are more electrons than holes on the NC due to the hole trapping process. Mathematically, the complete model for the 340 nm pump consists of a set of coupled differential equations

$$n_{\mathrm{exc}} = \min(n_{\mathrm{e}}, n_{\mathrm{h}}) \tag{S7}$$

$$\frac{dn_{\mathrm{e}}}{dt} = -\frac{n_{\mathrm{exc}}(n_{\mathrm{exc}}-1)}{2} k_{\mathrm{Auger}} - n_{\mathrm{exc}}(n_{\mathrm{e}} - n_{\mathrm{exc}}) \frac{k_{\mathrm{Auger}}}{2} + n_{\mathrm{e}}^{\mathrm{Auger}} k_{\mathrm{cool}} \tag{S8}$$

$$\frac{dn_{\mathrm{h}}}{dt} = -\frac{n_{\mathrm{exc}}(n_{\mathrm{exc}}-1)}{2} k_{\mathrm{Auger}} - n_{\mathrm{h}} k_{\mathrm{trap}} + n_{\mathrm{h}}^{\mathrm{Auger}} k_{\mathrm{cool}} \tag{S9}$$

$$\frac{dn_{\mathrm{e}}^{\mathrm{Auger}}}{dt} = \frac{n_{\mathrm{exc}}(n_{\mathrm{exc}}-1)}{2} k_{\mathrm{Auger}} + n_{\mathrm{exc}}(n_{\mathrm{e}} - n_{\mathrm{exc}}) \frac{k_{\mathrm{Auger}}}{2} - n_{\mathrm{e}}^{\mathrm{Auger}} k_{\mathrm{cool}} \tag{S10}$$

$$\frac{dn_{\mathrm{h}}^{\mathrm{Auger}}}{dt} = \frac{n_{\mathrm{exc}}(n_{\mathrm{exc}}-1)}{2} k_{\mathrm{Auger}} - n_{\mathrm{h}}^{\mathrm{Auger}} k_{\mathrm{cool}} \tag{S11}$$

$$\frac{dT_{\mathrm{lattice}}}{dt} \propto n_{\mathrm{e}}^{\mathrm{Auger}} k_{\mathrm{cool}} + n_{\mathrm{h}}^{\mathrm{Auger}} k_{\mathrm{cool}} \tag{S12}$$

where $n_h k_{trap}$ and $n_{exc}(n_e - n_{exc})\frac{k_{Auger}}{2}$ are the new terms in the kinetic model that were not present for the 510 nm (i.e. low energy) pump. The only new fitting parameter is k_{trap} which was fit in order to quickly trap the correct number of holes such that the overall temperature change is in agreement with the experiments. Importantly, the rest of the parameters are identical to those used in the modeling of the ultrafast electron diffraction data with the low energy, 510 nm pump and the time-resolved photoluminescence data. Excellent agreement between this revised model (Eqs. S7-S12), which considers hot hole trapping, and the experimental data is shown in main text Figure 3D. The time dynamics for the 340 nm pump are also summarized pictorially in Supplementary Figure 12.

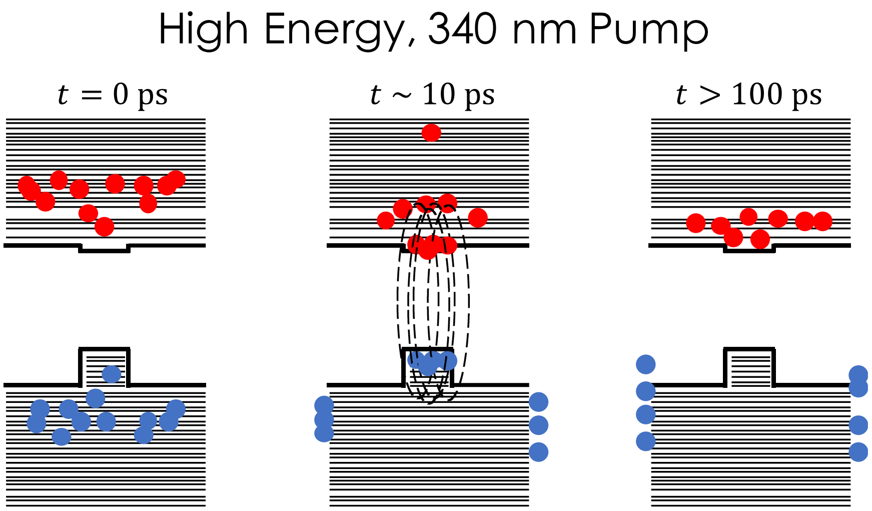

Supplementary Figure 12. Schematic representation of the initial state (left), an intermediate state (middle), and final state (right) of the kinetic model for the high energy, 340 nm pump. The intermediate state shows that a high energy electron has been generated by an Auger recombination event and that many of the initial holes have been trapped on the CdS surface resulting in general lattice heating and localized structural disorder, respectively.

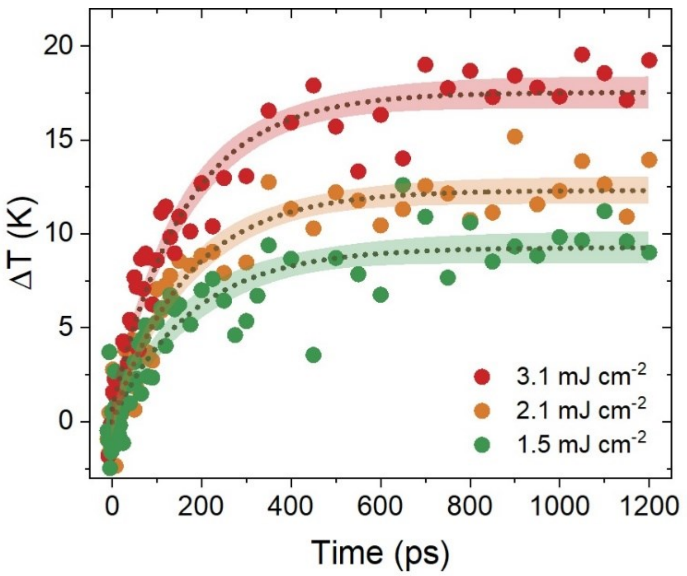

Supplementary Figure 13. Exponential fit to $\Delta T(t)$ in CdSe/CdS sample excited at 510 nm. Single exponential lifetimes are 178 ± 30 ps, 168 ± 20 ps and 162 ± 15 ps for excitation fluences of 1.5, 2.1 and 3.1 mJ cm^{-2}, respectively.

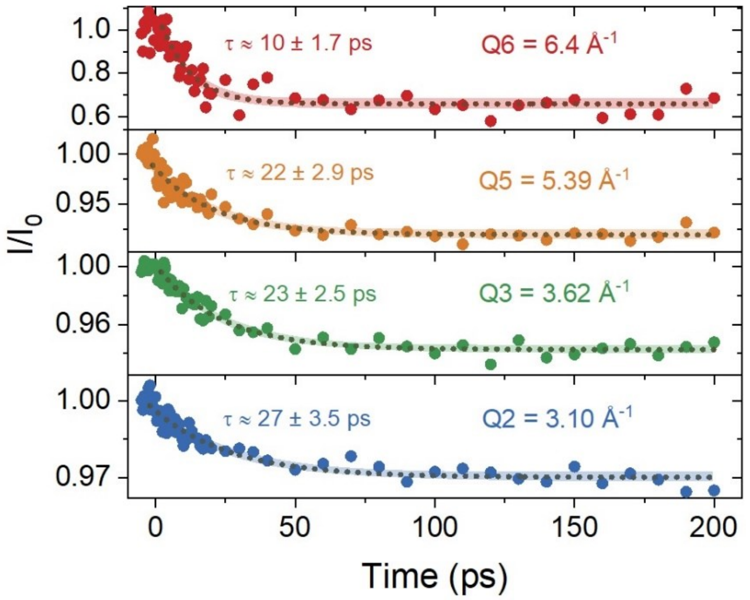

Supplementary Figure 14. $I(t)/I_0$ at four different diffraction peaks with 340 nm excitation. Single exponential lifetimes are displayed on the panels.

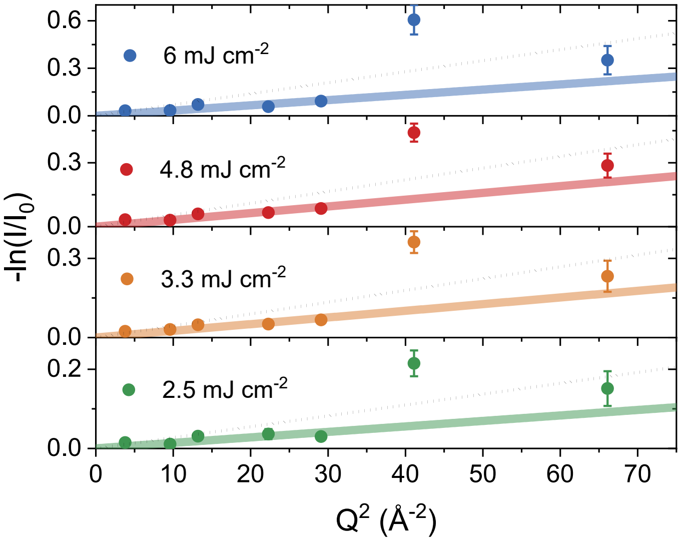

Supplementary Figure 15. $-\ln(I(t)/I_0)$ as a function of Q^2 under 340 nm excitation at 200 ps for fluences of 6.0, 4.8, 3.3, and 2.5 mJ cm^{-2}. Each excitation fluence displays a linear response at low Q (<30 Å^{-2}) and additional localized disorder at high Q (>40 Å^{-2}). Dotted lines show the linear fit to the whole range. Colored lines show the linear fit to the lowest five Q peaks. The error bars show standard error.

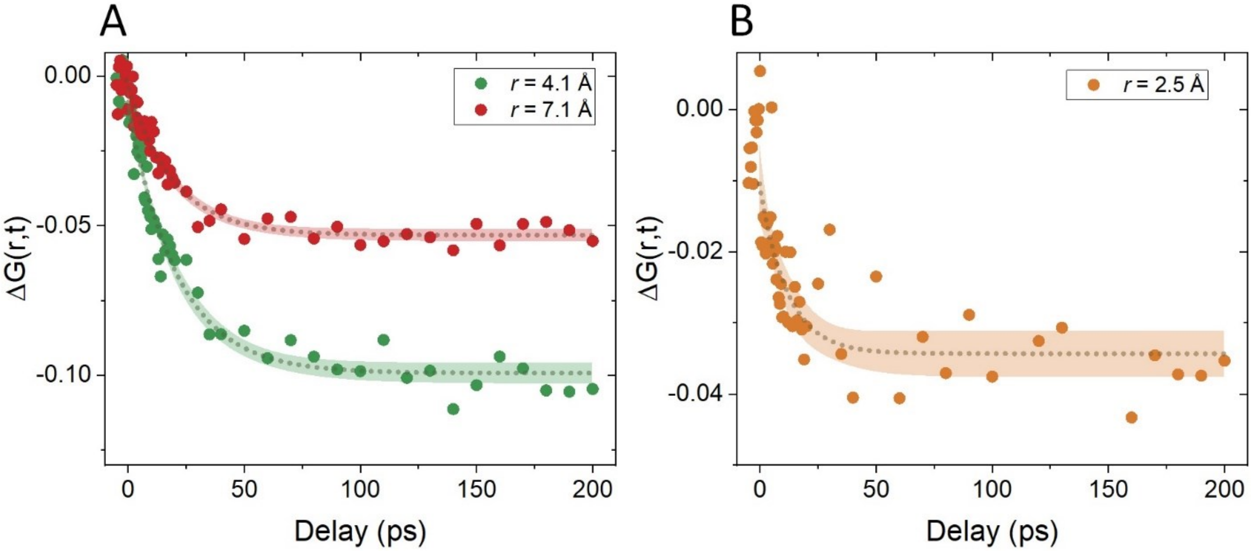

Supplementary Figure 16. (A) $\Delta G(r,t)$ measured at the peak of atomic correlations at r = 4.1 and 7.1 Å. They exhibit almost the same phenomenological time constant of 20 ps, consistent with overall heating of the particle, suggesting mainly a thermal response and minimal contribution from localized disorder at longer range correlations. (B) $\Delta G(r,t)$ measured at the peak of atomic correlation at r = 2.5 Å, which shows faster dynamics with a phenomenological time constant of 11 ps, indicative of the contribution of localized disorder to the first nearest neighbor peak.

Section G. Calculation of the electron and hole Auger channels in the core/shell CdSe/CdS nanocrystals

The lack of (or weak) signal of hole trapping in the low energy (510 nm) pump data for the CdSe/CdS core/shell quantum dots suggests that Auger recombination induced hole trapping is uncommon in these core/shell quantum dots. This finding is in contrast to the low energy pump data for the CdSe core only quantum dots that does show appreciable hole trapping occurring on the Auger recombination lifetime (τ_{AR}) timescale.

In order to understand and (partially) explain these findings, we analyzed the rates for generating a high energy electron ($\tau_{\mathrm{AR,e}}$) and high energy hole ($\tau_{\mathrm{AR,h}}$) via an Auger recombination event. These two Auger recombination channels are termed the electron and hole channel, respectively, and are shown schematically in Supplementary Figure 17.

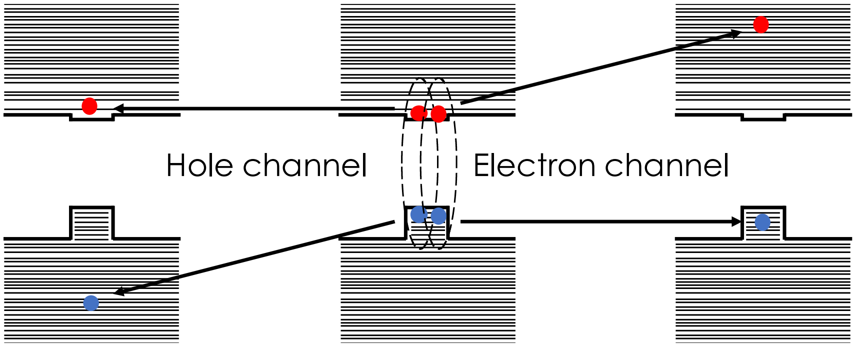

Supplementary Figure 17. Schematic representation of the electron and hole channels in Auger recombination. The initial biexcitonic state is shown in the middle as two excitonic states (dashed ovals). Electrons (holes) are shown as red (blue) circles. The electron (hole) channels shows that an electron (hole) receives a majority of the energy from exciton that recombines during the Auger recombination event.

The sum of the rates of these two channels equals the biexciton Auger recombination rate.

$$\tau_{\mathrm{AR}}^{-1} = \tau_{\mathrm{AR,e}}^{-1} + \tau_{\mathrm{AR,h}}^{-1}$$

The Auger recombination lifetimes for the specific channels can be calculated using

$$\tau_{\mathrm{AR,e}}^{-1} = \frac{2\pi}{\hbar Z_B}\sum_{B} e^{-\beta E_B}\sum_{a,i}\left|\sum_{b,c,k} c_{b,i}^{B}\, c_{c,k}^{B} V_{back}\right|^2 \delta(E_B - \varepsilon_a + \varepsilon_i)$$

and

$$\tau_{\mathrm{AR,h}}^{-1} = \frac{2\pi}{\hbar Z_B}\sum_{B} e^{-\beta E_B}\sum_{a,i}\left|\sum_{j,c,k} c_{a,j}^{B}\, c_{c,k}^{B} V_{ijck}\right|^2 \delta(E_B - \varepsilon_a + \varepsilon_i)$$

where Z_B is the partition function for the initial biexcitonic states taken at room temperature, E_B is the energy of the initial biexcitonic state, $\varepsilon_{a(i)}$ is the energy of the electron (hole) state in the final single excitonic state, $c^B_{a,i}$ are coefficients for the low energy excitonic states in the initial state that are obtained by solving the Bethe-Salpeter equation, and V_{rsut} is the Coulomb coupling. This method, and its stochastic implementation, have recently been described in detail and shown to predict quantitatively accurate Auger recombination lifetimes for core only quantum dots, core/shell quantum dots, nanorods, and nanoplatelets[19–21].

Using this method, we calculated these lifetimes for a CdSe/CdS core/shell quantum dot with a core diameter of 3.8 nm and 4 monolayers of a CdS as a shell and a 3.8 nm diameter CdSe quantum dot core without a CdS shell. We found that the electron and hole channels are approximately equal in the core only particle ($\tau_{\mathrm{AR,e}} \approx \tau_{\mathrm{AR,h}} \approx 100$ ps). On the other hand, the electron channel is significantly faster in the CdSe/CdS core/shell quantum dot. Specifically, we calculated the electron channel to have a lifetime of 280 ps compared to a lifetime of 967 ps for the hole channel in the aforementioned CdSe/CdS core/shell quantum dot.

Thus, our calculations indicate that the majority (approximately 75%) of hot carriers produced by Auger recombination events in the core/shell quantum dots will be electrons. This finding that the generation of hot holes via Auger recombination in core/shell quantum dots is relatively unlikely partially explains the lack of hole trapping signal in the 510 nm pump data for the CdSe/CdS core/shell quantum dot.

Section H. Calculation of the localized surface distortion magnitude

To estimate the localized distortion magnitude associated with the hot hole surface trapping, we compare transiently induced signals in the atomic pair distribution function under both 510 nm and 340 nm excitation conditions. Supplementary Figure 18A shows the transient atomic pair distribution function $\Delta G(r,t)$ measured with 510 nm (fluence of 2.1 mJ cm^{-2}) in the 8 ML core/shell sample (same data as in Fig. 2C of the main text). We use this case to calibrate the change in $\Delta G(r,t)$ to that of the induced mean squared atomic displacements ($\langle \Delta u(t)^2 \rangle$). In support of this, the dips observed in $\Delta G(r,t)$ at the atomic correlation peaks (Supplementary Figure 18) change linearly with the excitation fluence.

In the case of 510 nm excitation, we see that $\Delta G(2.5\ \text{Å}, t = 1000\ ps) = -0.0157$ and $\Delta G(4.1\ \text{Å}, t = 1000\ ps) = -0.0475$. The magnitudes of these dips are associated with the thermally induced $\langle \Delta u(t)^2 \rangle = 0.0028\ \text{Å}^2$. The induced $\langle \Delta u(t)^2 \rangle$ is valid for all the unit cells of the NC since we showed that the nanocrystal lattice homogenously heats with 510 nm excitation (Fig. 2 of the main text).

In the case of 340 nm excitation (Supplementary Figure 18B), we show $\Delta G(r,t)$ at early times (t = 5 ps) to extract out the localized disordering effect, as the thermal effect is slow in this case and evolves with a time constant of ~20 ps whereas localized disordering proceeds with a time constant of 3.5 ps. Consistent with the localized disordering, we see that the dip at the first nearest neighbor (2.5 Å) is significantly larger than others. By using the empirical knowledge gained from the response of $\Delta G(r,t)$ under pure transient heating (Supplementary Fig. 17A), we now subtract the contribution of the thermal effect from that of the localized disorder at $r = 2.5$ Å (see green and black arrows for the localized disorder and thermal disorder, respectively in Supplementary Figure 18B). After subtracting the thermally induced signal, we obtain the "dip" amplitude due to localized disordering as $\Delta G(r = 2.5\ \text{Å}, t = 5\ ps) = -0.009$.

If this disorder existed throughout the whole nanocrystal volume, then we estimate $\langle \Delta u(t)^2 \rangle = 0.0028 \times \frac{0.009}{0.0157} = 0.00161$ Å^2 by comparing its amplitude to the dip observed with 510 nm case. However, the localize disordering exists only at limited fraction of the nanocrystal as it arises from hot hole trapping on nanocrystal surfaces. To estimate the magnitude of the localized distortion, we consider the exciton density generated in the case of 340 nm excitation, which is $\langle N \rangle = 200$ under 4.8 mJ cm^{-2}. In addition, our kinetic model predicts that ~80% of the hot holes are trapped. Thus, we estimate that 160 hot holes are trapped. We assume that each trapped hot hole is localized to a single wurtzite CdS unit cell. From this, we can estimate the atomic mean squared displacement ($\langle \Delta\delta^2 \rangle$) of the localized distortion by comparing to the estimate of $\langle \Delta u(t)^2 \rangle$ for the whole nanocrystal getting affected. The volume of the NC (8 ML core/shell) is ~288.7 nm^3. Thus, it consists of in total ~2300 wurtzite CdS(Se) unit cells. We find the mean squared atomic displacement for a single localized disorder to be $\langle \Delta\delta^2 \rangle = \langle \Delta u(t)^2 \rangle \times \frac{2300\ unit\ cells}{160\ unit\ cells} = 0.023$ Å^2. Root mean squared atomic displacement associated with hot hole surface trapping event is then $\sqrt{\langle \Delta\delta^2 \rangle} = 0.15$ Å, which indicates the extent of the localized holes on the nanocrystal surface. It is comparable to previously reported small polaron size in other materials[22].

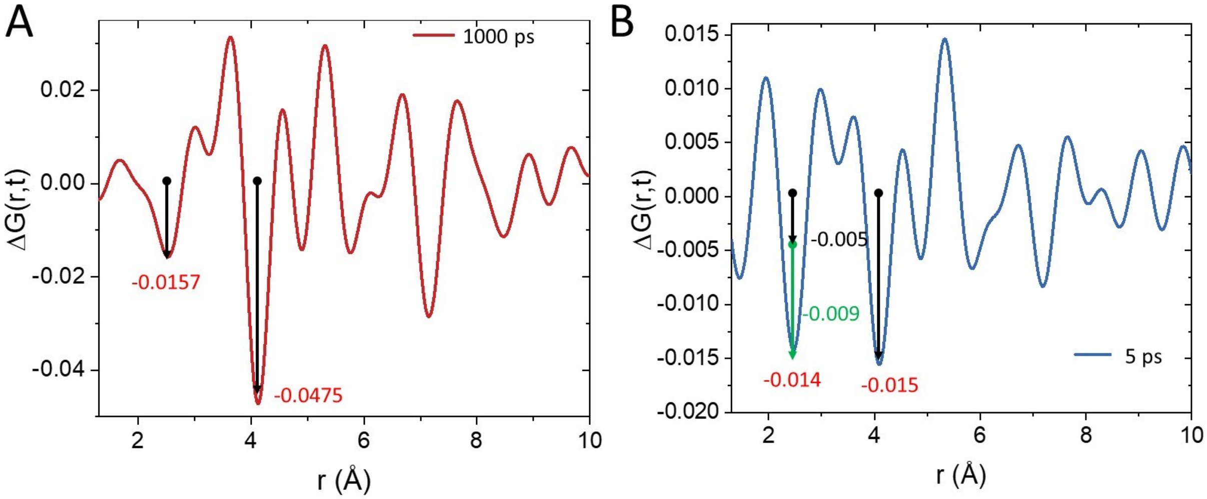

Supplementary Figure 18. Transient atomic pair distribution function calculated for (A) 510 nm excitation at later times (~1000 ps) when the nanocrystals is thermally equilibrated and (B) 340 nm excitation at early times (5 ps) before the thermal equilibration. In (B) the dominant signal comes from the localized disordering, which affects the first atomic pair correlation, and partial transient heating. We decouple the amplitude of the disorder caused on the first atomic correlation just from localized disordering (green arrow) by subtracting the effect of thermally induced response (black arrows).

Section I. Note on the local geometry of the surface small polarons

The spatial extent and local configuration of the surface hole polaron depend on the local symmetry, bonding and orbital structure. Thus, it is complicated to precisely estimate the true nature of the small surface polarons. Prior theory works have predicted that charges in various nanocrystals (e.g., CdS or metal oxides) may be localized to form polarons lying within particular atomic planes[23,24]. This would favor distortions along particular crystallographic directions. In this picture, we believe that the deviation starting with the Q6 peak can be understood by considering the relevant diffraction peaks underlying each

of the first six peaks Q1-Q6. Supplementary Table 1 is showing our indexing of the Miller indices (hkl) of these peaks.

One notes that Q6 is the only peak involving large l indices (e.g., (215) and (116)), corresponding to lattice planes oriented perpendicular to the c-axis of the wurtzite unit cell of the nanocrystals. Thus, the deviation in peak Q6 from the linear response associated with the isotropic heating is indicative of unit cell distortions occurring favorably along the c-axis, which are amplified by the large l in the structure factor ($F_{hkl} = \sum_n f_n e^{2\pi i(hx_n+ky_n+lz_n)}$). In this model one may potentially understand in a self-consistent way why it is that peaks Q1 and Q3 lie above the linear fit while Q2 lies below (as is the case for all fluences shown above in Fig. S15) since Q2 in particular corresponds to a set of planes with l=0, aligned parallel to the c-axis with structure factor independent of distortions along the c-axis. In summary the sharp deviation of peak Q6 from the linear Debye-Waller-like response can be understood as indicative of the development of short-range distortions likely involving significant c-axis displacements within the wurtzite unit cell of the nanocrystal. Higher Q-resolution measurements in the future could potentially shed further light on the atomic distortions involved.

References:

1. Hanifi, D. A. *et al.* Redefining near-unity luminescence in quantum dots with photothermal threshold quantum yield. *Science* **363**, 1199–1202 (2019).

2. Weathersby, S. P. *et al.* Mega-electron-volt ultrafast electron diffraction at SLAC National Accelerator Laboratory. *Review of Scientific Instruments* **86**, 73702 (2015).

3. Guzelturk, B. *et al.* Nonequilibrium Thermodynamics of Colloidal Gold Nanocrystals Monitored by Ultrafast Electron Diffraction and Optical Scattering Microscopy. *ACS Nano* **14**, 4792–4804 (2020).

4. Gates-Rector, S. & Blanton, T. The Powder Diffraction File: a quality materials characterization database. *Powder Diffraction* **34**, 352–360 (2019).

5. Wu, X. *et al.* Light-induced picosecond rotational disordering of the inorganic sublattice in hybrid perovskites. *Science Advances* **3**, e1602388 (2017).

6. Billinge, S. Chapter 16 Local Structure from Total Scattering and Atomic Pair Distribution Function (PDF) Analysis. in *Powder Diffraction: Theory and Practice* 464–493 (The Royal Society of Chemistry, 2008). doi:10.1039/9781847558237-00464.

7. Peng, L. M. Electron Scattering Factors of Ions and their Parameterization. *Acta Crystallographica Section A: Foundations of Crystallography* **54**, 481–485 (1998).

8. Plimpton, S. Fast parallel algorithms for short-range molecular dynamics. *Journal of Computational Physics* **117**, 1–19 (1995).

9. Grünwald, M., Zayak, A., Neaton, J. B., Geissler, P. L. & Rabani, E. Transferable pair potentials for CdS and ZnS crystals. *Journal of Chemical Physics* **136**, 234111 (2012).

10. Gao, H. X. & Peng, L.-M. Parameterization of the temperature dependence of the Debye-Waller factors. *Acta Crystallographica Section A* **55**, 926–932 (1999).

11. Tewary, V. K. & Yang, B. Singular behavior of the Debye-Waller factor of graphene. *Physical Review B* **79**, 125416 (2009).

12. Straub, J. E., Borkovec, M. & Berne, B. J. Calculation of Dynamic Friction on Intramolecular Degrees of Freedom. *J. Phys. Chem* **91**, 4995–4998 (1987).

13. Egorov, S. A., Rabani, E. & Berne, B. J. Nonradiative relaxation processes in condensed phases: Quantum versus classical baths. *Journal of Chemical Physics* **110**, 5238–5248 (1999).

14. Bader, J. S. & Berne, B. J. Quantum and classical relaxation rates from classical simulations. *The Journal of Chemical Physics* **100**, 8359–8366 (1994).

15. Gillespie, D. T. Exact stochastic simulation of coupled chemical reactions. *The Journal of Physical Chemistry* **81**, 2340–2361 (1977).

16. Ben-Shahar, Y. *et al.* Charge Carrier Dynamics in Photocatalytic Hybrid Semiconductor–Metal Nanorods: Crossover from Auger Recombination to Charge Transfer. *Nano Letters* **18**, 5211–5216 (2018).

17. Hendry, E. *et al.* Direct Observation of Electron-to-Hole Energy Transfer in CdSe Quantum Dots. *Physical Review Letters* **96**, 57408 (2006).

18. Wu, K., Lim, J. & Klimov, V. I. Superposition Principle in Auger Recombination of Charged and Neutral Multicarrier States in Semiconductor Quantum Dots. *ACS Nano* **11**, 8437–8447 (2017).

19. Philbin, J. P. & Rabani, E. Auger Recombination Lifetime Scaling for Type I and Quasi-Type II Core/Shell Quantum Dots. *Journal of Physical Chemistry Letters* **11**, 5132–5138 (2020).

20. Ben-Shahar, Y. *et al.* Charge Carrier Dynamics in Photocatalytic Hybrid Semiconductor-Metal Nanorods: Crossover from Auger Recombination to Charge Transfer. *Nano Letters* **18**, 5211–5216 (2018).

21. Philbin, J. P. *et al.* Area and thickness dependence of Auger recombination in nanoplatelets. *Journal of Chemical Physics* **153**, (2020).

22. Billinge, S. J. L., DiFrancesco, R. G., Kwei, G. H., Neumeier, J. J. & Thompson, J. D. Direct Observation of Lattice Polaron Formation in the Local Structure of $La_{1-x}Ca_xMnO_3$. *Phys. Rev. Lett.* **77**, 715–718 (1996).

23. Carey, J. J. & McKenna, K. P. Does Polaronic Self-Trapping Occur at Anatase TiO_2 Surfaces? *Journal of Physical Chemistry C* **122**, 27540–27553 (2018).

24. Cline, R. P., Utterback, J. K., Strong, S. E., Dukovic, G. & Eaves, J. D. On the Nature of Trapped-Hole States in CdS Nanocrystals and the Mechanism of Their Diffusion. *Journal of Physical Chemistry Letters* **9**, 3532–3537 (2018).
